# Self-diagnosis and self-medication based on internet search among Non-Medical University students of Karachi

**DOI:** 10.1097/MS9.0000000000002605

**Published:** 2024-10-01

**Authors:** Adarsh Raja, Shafin Bin Amin, Bazil Azeem, Sandesh Raja, Yusra Aftab, Maham Rafi, Fnu Abheman, Kumar Sukhani, Piyasi Mal, Noor Ul-Ain, Fazal Manan, Rabbia Aqeel, Hamza Rahat, Pervaiz Ali, Naresh Kumar, Kiran Khan, Varsha Sharma

**Affiliations:** a Shaheed Mohtarma Benazir Bhutto Medical College Lyari, Karachi, Pakistan; b Dow Medical College, Dow University of Health Sciences, Sindh, Pakistan; c Associate Professor, Department of Community Medicine, Shaheed Mohtarma Benazir Bhutto Medical College Lyari, Karachi, Pakistan; d Department of Internal Medicine, Nepal Medical College, Kathmandu, Nepal

**Keywords:** internet, on-medical universities, self-diagnosis, self-medication

## Abstract

**Background:**

For a decade, the topic of self-diagnosis and self-medication has gained significant attention due to the widespread availability of information on the internet and over-the-counter medication. This research explores the rational considerations behind individuals’ self-diagnosis and self-medication practices. Our main objective is to find out the frequency of self-diagnosis and self-medication in the general population and its associated risks and benefits.

**Methods:**

A cross-sectional community-based prospective study was conducted over 7 months and included 160 students from various nonmedical universities in Karachi. A questionnaire regarding baseline characteristics, self-medication, and self-diagnosis was made, and the data was collected from the participants and then analyzed using SPSS statistical software.

**Results:**

One-fifth of the participants used the method of diagnosing themselves regularly, whereas 9% of the involved population demonstrated medicating themselves very often. However, most of the population had self-diagnosed (50.6) or self-medicated (61.9) sometimes. The internet was the primary source of searching (75%), and home remedies were the preferred medications (71.7%). The two primary reasons for this were the scarcity of time and resources.

**Conclusion:**

Overall, our study points out the significance of self-medication and self-diagnosis among different nonmedical students of Karachi. Teaching people about medicines, enforcing strong prescription policies, and providing medical facilities are vital steps toward preventing this problem. The role of doctors and medical students is significant; therefore, detailed doctor-patient communication needs to be encouraged.

## Introduction

Highlights

- 75% of nonmedical university students in Karachi have self-diagnosed, and 76.9% have self-medicated at least once.
- 75% of students rely on the internet for medical information, showing a significant dependence on online resources.
- 71.3% of students prefer using home remedies and previously prescribed medications for similar symptoms.
- Scarcity of time and financial resources are major reasons for self-diagnosis and self-medication.
- The study highlights the need for increased awareness, stricter prescription policies, and improved healthcare access to address these practices.

Nowadays, the world has become a global village. There is copious amounts of online information but no authentication^1–3^. Irrespective of age and sex, the general population relies on social media or available AI programs such as ChatGPT for most of the information, including academic and health information, and trusts it without doing any research^4,5^. Instead of going to the doctor or asking an expert, they find it convenient and cheaper to look for information online^6,7^. Self-diagnosis is the process of diagnosing or identifying medical conditions in oneself. It may be assisted by medical dictionaries, books, resources on the internet, past personal experiences, or recognizing symptoms or medical signs of a condition that a family member previously had^8^. It is a process where individuals observe within themselves symptoms of pathology and identify a disease or disorder without medical consultation^9^. According to the WHO definition, self-medication involves the use of medical products by the consumer to treat self-diagnosed disorders or symptoms or the intermittent or continued use of medication prescribed by a physician for chronic or recurrent diseases or symptoms^10^. On average, the prevalence of people searching for information about their medical condition is almost 15.49%^11^.

Moreover, the prevalence of self-medication in developing countries widely varies between 12.7 and 95%^12,13^. Some common examples of self-medication are using drugs for depression, using drugs to calm bipolar disorders, using drugs to alleviate physical pain, using drugs to improve one’s mood, and using drugs to ease anxiety^11^. There are numerous causes of self-medication, which include attempting to deal with past trauma, addressing social anxiety, boredom, coping mechanisms for mental health conditions, changing certain habits, or deliberately influencing moods https://www.aspenridgerecoverycenters.com/causes-of-self-medication. Likewise, various medications are commonly used for this purpose, which include antipyretics (47.62%), analgesics (23.81%), antibiotics (19.05%), and anticold (9.52%)^14^. Self-medication is associated with multiple risks, such as pre-existing conditions that can worsen when the diagnosis is incorrect, delay in seeking appropriate consultation, increased risk of drug resistance and dependence, and masking of severe diseases^12,13,15^. Nowadays, self-diagnosis and self-medication through internet search is very common, but it is not valid^15^; one should always consult a professional opinion to help determine the cause of one’s disease. Also, self-diagnosis cannot give access to the treatment as the medications need to be confirmed by a professional person that complies best with the person’s health status, does not interact with any other medication that a person is using, and does not worsen any of his other health conditions or diseases. This research aims to explore the rationale considerations behind the individuals engaging in self-diagnosis and self-medication practices. The study will investigate the motivations, beliefs, and decision-making processes that led individuals to self-diagnose their medical conditions and subsequently self-medicate. It will analyze the potential benefits and risks associated with these practices, considering factors such as self-diagnosis accuracy, appropriateness of self-medication, and possible adverse effects.

## Methods

### Participants and sampling

A cross-sectional study was conducted during the year 2023 between April 2023 and November 2023 among the students of general or nonmedical universities of Karachi. The Community Medicine Department of the College approved this research study. The involvement of sample size was performed through an open epi sample size calculator with a 5% margin error and 95% CI. There was a total of 160 students, including both sexes. The process of free sampling was employed. The inclusion criteria of this study encompassed students who were enrolled in nonmedical universities in Karachi. Self-diagnosis was defined as diagnosing or identifying medical conditions in oneself.

On the other hand, self-medication involves the use of medical products by the consumer to treat self-diagnosed disorders or symptoms or the intermittent or continued use of medication prescribed by a physician for chronic or recurrent diseases or symptoms. Meanwhile, the students who did not show interest, did not consent, belonged to medical universities, or were not from Karachi were excluded. Proper authorization was obtained from the participants, and the work was reported to be in line with the strengthening the reporting of cohort, cross-sectional, and case–control studies in surgery (STROCSS) criteria^16^.

### Surveys and measures

Data was collected through questionnaires and distributed through various online platforms, including WhatsApp groups, Instagram, and various other social media platforms; this aimed to include participants with different backgrounds. Some students received the questionnaire through e-mails; however, confidentiality was not breached. Follow-up reminders were sent to participants to increase responses. The majority of the reactions were gained from Google Forms. Knowledge regarding the concept of self-medication and self-diagnosis was the main aim of these questionnaires. The questionnaires were adequately designed to avoid confusion and wrong feedback. The questionnaires were adjusted to the context of the study. They ensured reliability, validity, and accuracy through an expert review, who then provided structured feedback regarding the conciseness and relevance of the questions. Additionally, data was pretested before implementation, and pretesting was performed to identify any ambiguities and difficulties and improve the study’s clarity.

### Statistical analysis

Statistical analysis was conducted using SPSS version 25.0. Descriptive statistics such as mean, SD, percentages, and dichotomous data were used to summarize the responses from the participants. For the assessment of data regarding self-medication and self-diagnosis based on internet search, *χ* ^2^ tests were used, and *P*<0.05 was considered significant. Other validity analyses, such as content and construct validity analysis, are also used.

### Ethical approval

The research study on self-medication and self-diagnosis based on an internet search among nonmedical universities in Karachi was approved by the Community Medicine Department of the College. Students were told that their data would remain confidential and that we would use it only for a research study. They can withdraw at any time during or before the study. Access to reported data was only available to the team leader and was stored on secure servers. Confidentiality and data security were maintained throughout the study by anonymizing data. Instead of mentioning original names, unique codes were employed. The data was encrypted in transit.

## Results

### Participants demographics

**Figure 1:**
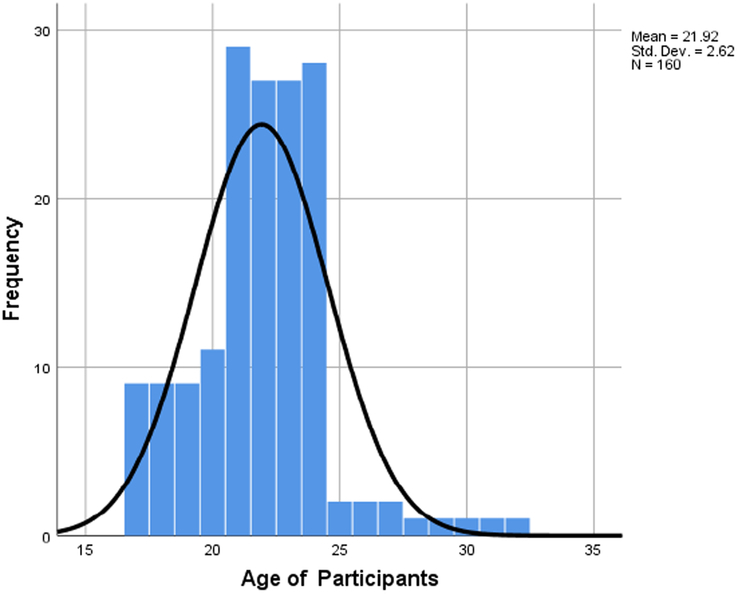
Histogram representing the age of participants.

**Figure 2:**
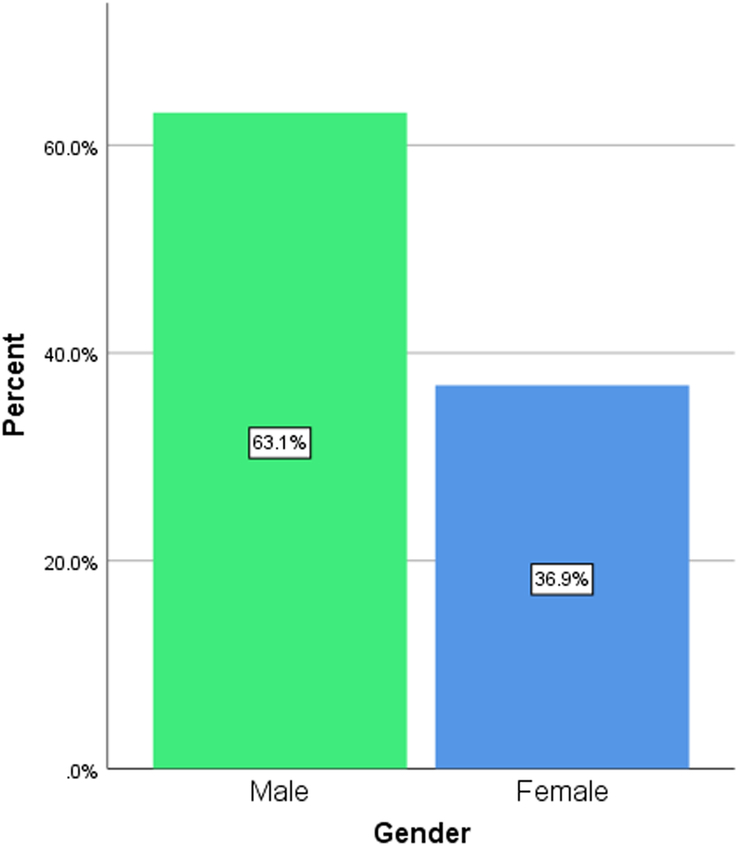
Bar graph representing the sex of the participants.

**Figure 3:**
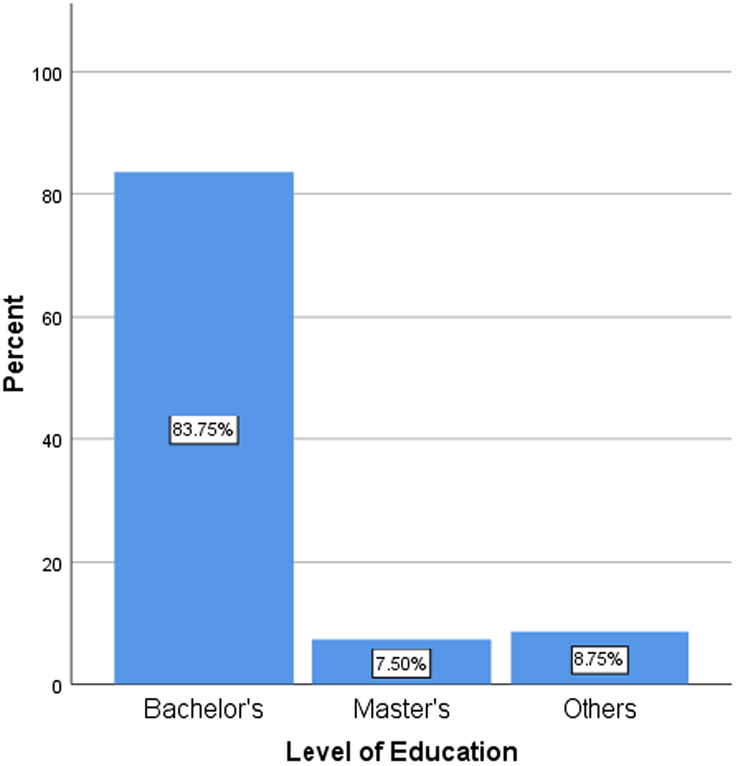
The educational qualification of the participants.

**Figure 4:**
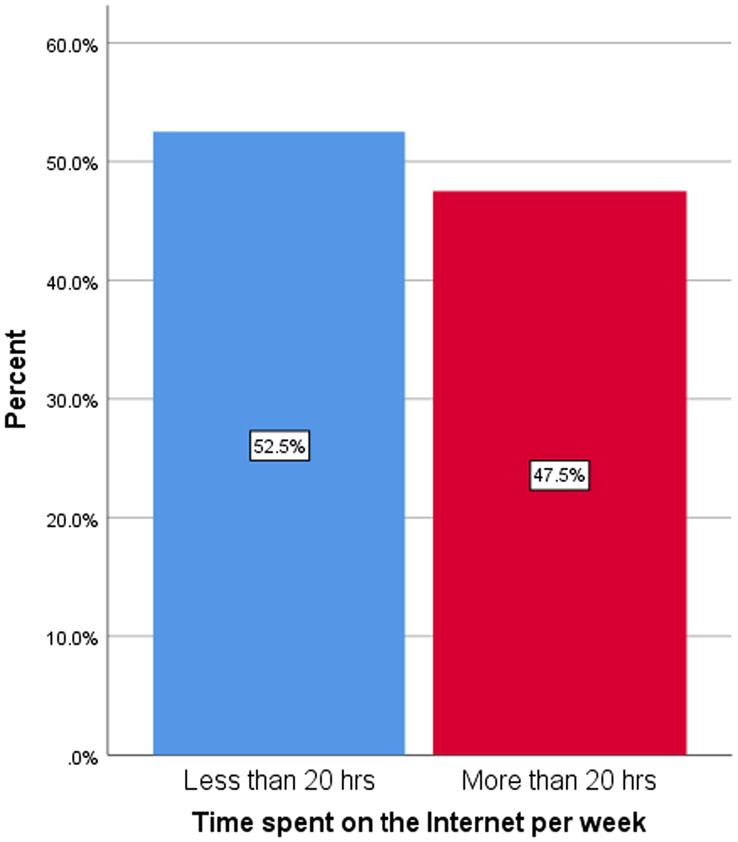
Bar graph representing the average amount of time spent on the internet by the participants.

A total number of 160 students (*N*=160) participated in the study. The mean age was 21.92 (SD=2.62), of which males account for 63.1 and females 36.9% of the total sample size (Figs 1 and 2). The educational background of the participants based on their level of graduation, that is bachelor’s (83.75%), master’s (7.50%), and others (8.75%), is denoted by the bar graph in Figure 3. The participants included had a nonmedical university background based in Karachi, Pakistan. Our research included students from nonmedical universities in Karachi, such as NED University, University of Karachi, Iqra University, and Allama Iqbal Open University. The students who participated in our research came from different fields of study, including business administration, architecture, LLB, software engineering, computer science, etc. Figure 4 represents the average amount of time consumed by the participants while browsing the internet per week. 52.5% of the participants averaged <20 h per week, and 47.5% averaged >20 h per week.

#### Self-diagnosis

The frequency of students involved in self-diagnosis by searching for their symptoms on the internet is shown in Table 1. 5.6% of the participants searched for their symptoms all the time, 18.8% often, 50.6% sometimes, and 25% had never carried out this practice. The medical conditions that are most commonly self-diagnosed are anxiety, common flu, chicken pox, hair fall, dandruff, diabetes, malaria, stomach issues, asthma, depression, acne, dengue, and migraine. In summary, a great deal of (75%) participants had self-diagnosed themselves more than once in their lifetime.

**Table 1:** Shows the frequency of participants that self-diagnose themselves by searching for it on the internet.

How often do you use the Internet to search about your symptoms and diagnose yourself?			
All the time	Often	Sometimes	Never
9	30	81	40
5.60%	18.80%	50.60%	25.00%

#### Self-medications

Regarding self-medication, 5.6% of the participants had self-medicated themselves almost all the time, 61.9% sometimes, 9.4% had tried it once, and 23.1% had never self-medicated. A total of 76.9%participants have self-medicated. Table 2 shows that more students are involved in self-medication (73.9%).

**Table 2:** Shows the frequency of participants that self-medicate.

Have you ever self-medicated?			
All the time	Once	Sometimes	Never
9	15	99	37
5.60%	9.40%	61.90%	23.10%

#### Reasons for self-medication and self-diagnosis

Regarding the reasons that led the participants to self-medication and self-diagnosis, a series of questions were included in the questionnaire (Table 3). The key components that were inquired about in the questionnaire were internet usage to search for symptoms and diagnosis (75%), taking medicines because they were prescribed by the doctor before for similar symptoms (71.3%), preferring home remedies that one sees on the internet (71.3%), avoid visiting the doctor due to lack of resources (53.7%), or/and visiting a doctor is time-consuming (49.4%).

**Table 3:** Reasons that lead people to self-diagnose and self-medicate.

Questions	Response	Count	Column *N* %
How often do you use the internet to search symptoms and diagnose yourself?	All the time	9	5.60
	Often	30	18.80
	Sometimes	81	50.60
	Never	40	25.00
Have you ever taken medication without prescription because you were prescribed them before?	All the time	8	5.00
	Often	19	11.90
	Sometimes	87	54.40
	Never	46	28.70
Instead of visiting a doctor, do you prefer home remedies that you see on the internet?	All the time	19	11.90
	Often	20	12.50
	Sometimes	75	46.90
	Never	46	28.70
Do you avoid visiting a doctor due to lack of resources?	All the time	15	9.40
	Often	13	8.10
	Sometimes	58	36.30
	Never	74	46.30
Do you avoid visiting a doctor because it is time-consuming?	All the time	14	8.80
	Often	15	9.40
	Sometimes	50	31.30
	Never	81	50.60

#### Side-effects of self-medications

When asked about experiencing side-effects after self-medication, 2.5% of the participants had side-effects all the time, 7.5% once, 15% sometimes, and 75% never experienced any side-effects (Table 4). The most experienced adverse effects are rashes, fatigue, vomiting, sleep disturbance, GI upset, etc. Table 5 shows that 10% of the participants visited the doctor after experiencing side-effects, 8.1% once, and 13.1% had visited a fair number of times.

**Table 4:** Shows the frequency of people who have experienced side-effects after self-medication.

Have you ever experienced side-effects from self-medication?			
All the time	Once	Sometimes	Never
4	12	24	120
2.50%	7.50%	15.00%	75.00%

**Table 5:** Shows the frequency of people who visited the doctor after experiencing side-effects from self-medication.

Have you ever visited a doctor after facing the worst symptoms of self-medication?			
All the time	Once	Sometimes	Never
16	13	21	110
10%	8.10%	13.10%	68.80%

#### Safety of self-medications

Finally, the participants were asked whether they consider self-medication safe (Table 6). 16.3% answered positively, 31.3% responded negatively, and 52.5% were unsure about the consequences.

**Table 6:** Shows the number of participants that considered self-diagnosis and self-medication as correct or incorrect.

Do you believe self-medication is safe?		
Yes	No	Maybe
26	50	84
16.30%	31.30%	52.50%

#### Future of self-medication and self-diagnosis

Lastly, the participants were asked whether or not they would practice self-medication in the future (Table 7). 16.3% of the participants answered Yes, 27.5% refused, 40.6% were unsure, and 15.6% could not predict the future.

**Table 7:** Shows the frequency of participants that will self-diagnose and self-medicate in the future.

Will you self-diagnose and self-medicate in future too?			
Yes	No	Maybe	Don’t know
26	44	65	25
16.30%	27.50%	40.60%	15.60%

## Discussion

We conducted this study to show the role of the internet in diagnosing and treating one’s medical problem. This study was needed due to the extensive nature of our internet and social media, which play a crucial role in this condition. The result of our research showed that 76.9% of participants have self-medicated, and 75% of participants have self-diagnosed themselves once in their lifetime, which was mainly due to a scarcity of resources. However, 75% of the participants never experienced any side-effects. The Internet seems to be the primary source of information in such cases.

Additionally, uncertainty was observed within the participants when they were asked about using these methods in the future. We cautiously approached the evidence, enhancing our study’s quality. Overall, our study highlights the importance of the internet in self-diagnosis and self-treatment, the reasons behind using these methods, and the disadvantages.

These practices of self-diagnosing and self-medication are pretty standard throughout the globe. A cross-sectional study conducted in Indonesia demonstrated that for common conditions like cold, flu, etc., parents often diagnose and give medications to their children without proper consultation^17^. Likewise, a survey was conducted in Wuhan, a city in China. ‘Cluster sampling’ method was used, which suggested the fact that if the disease is not severe enough, then people self-diagnose and self-medicate. The illnesses in this case were common cold and cough. 45.5% of the population used these methods because they knew medicine from their past experiences. In comparison (51.2%) of participants mentioned that because they had read about medications in magazines and on the internet, they felt it was unnecessary to visit the doctor.

In some participants, a lack of resources was observed, similar to our results^18^. However, in this modern era of development, the internet could also be used to refrain from such situations. The development of ChatGPT’s and AI bots has created an impact on enhancing general awareness and provides an excellent level of knowledge. A study was conducted on this subject with the purpose of showing whether this development of technology is beneficial or harmful. This effect was observed through various questions. Ultimately, it showed that ChatGPT increased the depth of knowledge among the participants, leading to believe that it could be a key factor in the prevention of self-medication and self-diagnosis in the future^5^. Apart from easy access to the internet, easy access to medications and lack of time also plays a crucial role in this problem. One cross-sectional study conducted in the Faisalabad district showed that people usually adapt to these methods because of easy availability and lack of time. However, the preferred drugs in this case were analgesics, antipyretics, and NSAIDs^19^. Financial reasons are also potential parameters when it comes to self-diagnosis and management. Lack of finances affects the ability to achieve proper management and, thus, results in the avoidance of hospitals, making it more cost-effective to manage at home^18–20^. Another factor that promotes these concepts is the improper adoption of prescription policies. Studies show that pharmacists often sell antibiotics without prescriptions^21^. An observational study was conducted in Karachi, which showed that most drugs sold by pharmacists were antibiotics. However, analgesics were more common in terms of self-medication^22^. These findings coincided with the results of our study.

A study conducted in Faisalabad showed that because of a lack of time and financial problems, people engage in self-diagnosis and self-medication. Our study showed the relation between these factors and self-diagnosis and self-medication. Our study showed that males medicate and diagnose themselves more often as compared to females. Still, one study conducted in Saudi Arabia demonstrated that female students (61.9%) indulge in these activities more than males (38%). However, this study included participants from the medical profession^23^.

Similarly, another study was conducted, which demonstrated that medical students were vulnerable to adopting these methods, and females were at an increased risk^24^. Another study in Serbia showed that females (5.6%) treated themselves more than males (2.2%). However, this practice was seen more frequently in females of older age. As for males, unemployment was the main factor^25^. A cross-sectional study was performed in India, which also suggested that women had a higher chance of medicating themselves as compared to males. However, this was more common in uneducated females^26^.

Another concerning fact is that people consider it to be safe. Our study showed that 16.3% of participants thought it to be safe. Many studies indicate that people believe this is safe^16–18,27^. There appear to be various sources from which people extract information when diagnosing or treating themselves. A study on third-year medical students highlighted that most medical students use websites like Google and Meditech. Around 96.1% of people used Google Scholar to search for their diagnoses and medications^28^. A scoping review was done, which showed that young people are sensitive and easily rely on the information displayed on the internet^29^. A cross-sectional study was conducted in Islamabad in 2020, proving that people of lower income gather their information from online search engines.

In contrast, people from higher income groups go to the doctor. Another essential factor highlighted in this study was that using online sources worsened the diagnosis^30^. However, another study mentions that people with higher incomes or who possessed ample knowledge were the ones to use the internet^31^. However, since our participants were university students, we found no relation to this parameter in our research. Likewise, this parameter is also discussed with the findings of our study^32^. Our research and other studies in various contexts may help clarify the significance of self-diagnosis and self-medication based on internet research on people’s lives. Similar research can aid in determining the root cause of self-diagnosis and self-medication, as the adverse effects faced by engaging in self-diagnosis and self-medication, and, more importantly, raising awareness among people about whether self-diagnosis and self-medication are reliable or not.

Because of the easy availability of over-the-counter (OTC) medications, they were the significant culprits used regularly. Generally, they are considered safe; however, they can be addicting and can lead to various issues of the heart, kidneys, and brain. Concerns also arise when multiple of them are taken together, cross-reacting with each other and potentiating their effects^33^.

Our study provides insights regarding this topic. However, it has certain limitations. Firstly, due to the lack of response from medical professionals, we could not discuss the relationship of our findings. Consequently, it resulted in an absence of a control group. Secondly, a few participants were involved, and the nonrepresentative sample usage affected our results’ generalizability. Therefore, researchers with bigger and better sample sizes are required in future studies. Thirdly, a limited number of data resulted in an inability to discuss the implications of confounding variables, like health literacy, access to healthcare, socioeconomic status, etc.; future studies must mitigate these effects. Fourthly, this study was cross-sectional, making it prone to bias. Another limitation is the potential for selection bias due to the use of online platforms for data collection; this imposes and suggests using more rigorous methods to collect data in future studies. Moreover, using the control group in future studies may be beneficial as it makes it easy to determine if the findings are part of a broader trend. Also, a more relevant time frame, such as the past year, may give more robust and better results.

## Conclusion

Our research found that 75% of participants have self-diagnosed, and 76.9% have self-medicated once in their lifetime. The internet plays a crucial role in self-diagnosis; almost 75% of students use the internet for self-diagnosis and medication. The leading cause for self-diagnosis and self-medication among the participants was that they had been prescribed the same medications before for the same symptoms. Home remedies preferences, lack of financial resources, and time-consuming visits to a doctor were also the causes. Also, most of them were not quite sure about self-medication whether it was safe or not. This uncertainty indicates a lack of confidence in self-medication, likely due to limited knowledge about the risks involved. These all suggest that barriers to accessing professional healthcare might drive students toward self-diagnosis and self-medication as more convenient alternatives. Multiple things should be done which will lead to a reduction in this problem. The government of Pakistan should enforce strict policies regarding the prescription of drugs, and awareness should be provided to the people so that they are aware of the problems and their consequences.

Moreover, the cause of this problem needs to be addressed. Proper hospital facilities, short waiting times, and cost-effectiveness should be considered. Additionally, people should be provided with information about their medications through the doctor. Increasing awareness and improvement in healthcare access is also recommended with health literacy. Implementing all these telemedicine techniques to improve accessibility towards MAT (medication-assisted treatment) may be helpful, and regulatory barriers can be improved.

## Ethical approval

Ethics Committee: Shaheed Mohtarma Benazir Bhutto Medical College Lyari, Karachi Ref: SMBBMC/IRB/-20.

## Consent

Written informed consent was obtained from the patient for publication and any accompanying images. A copy of the written consent is available for review by the Editor-in-Chief of this journal on request.

## Source of funding

The authors received no extramural funding for the study.

## Author contribution

A.R. and S.B.A.: concept, software, manuscript writing, and editing; Y.A. and M.R.: methodology, manuscript writing, and editing; F.A., K.S., and P.M.: results, manuscript writing, and editing; N.-U.-A., F.M., R.A., and H.R.: discussion, manuscript writing, and editing; P.A. and N.K.: manuscript review, manuscript writing, and editing; K.K.: supervision, administration, manuscript writing, and editing; V.S.: supervision, administration, manuscript writing, and editing.

## Conflicts of interest disclosure

The authors declare that they have no known competing financial interests or personal relationships that could have appeared to influence the work reported in this paper.

## Research registration unique identifying number (UIN)

Our study is a retrospective cross-sectional analysis using pre-existing data, thus not requiring prior registration.

## Guarantor

Varsha Sharma.

## Data availability statement

The data sets are publicly available.

## Provenance and peer review

Not invited.
